# Reporting Race and Ethnicity in Population Health and Clinical Research From Japan

**DOI:** 10.2188/jea.JE20220244

**Published:** 2023-11-05

**Authors:** Soichiro Saeki, Misa Kusumoto

**Affiliations:** Center Hospital of the National Center for Global Health and Medicine, Tokyo, Japan

**Keywords:** clinical trial, epidemiologic studies, health equity, global health, social determinants of health

August 18 marked the first year after the new amendment to the *AMA Manual of Style* that addressed how race and ethnicity should be reported in medical and science journals, which was published as an editorial in *JAMA*.^1^ This amendment maintains uniformity and clarity in its wording, while promoting equity and fairness across race and ethnicity, as well as acknowledging that race and ethnicity are part of a social construct that should be reported along with other social demographic features and social determinants.^1^

Recently, there has been an upsurge in the reporting of race in medical journals, particularly in light of findings suggesting that outcomes from the novel coronavirus 2019 (COVID-19) disease vary by race and ethnicity.^2^ The “Black Lives Matter” movement^3^ and talks about prejudice towards Asian Americans^4^ in the United States have also sparked a desire to tackle discrimination from a variety of angles.^5^ Medicine and life science were not exceptions. However, recognizing racial and ethnic differences can perhaps result in discrimination. Hence, this amendment of the *AMA Manual of Style* to clarify race and ethnicity as one of the many social determinants of health aims to combat this situation.^1^

Such trends have led researchers to look into racial disparities in diseases other than COVID-19. For example, whereas sex differences in out-of-hospital cardiac arrest interventions have previously been reported, a recent study found that racial disparities were also evident in the interventions patients receive.^6^ This trend is expected to continue, given these features were not properly examined in many fields. The amendment in the *AMA Manual of Style* would serve as a foundation for studies to consider the influence of race and ethnicity for other healthcare settings.

However, reports on race and ethnicity have been few from Japanese studies. Many Japanese studies state that their studies were conducted on the “Japanese” but do not report on how they obtained such data. We believe that this is because most Japanese studies only include Japanese patients. This is caused by several factors. First, obtaining consent from patients who do not speak Japanese is difficult.^7^ Because Japanese is a rare language, patients who are not Japanese are less likely to speak it as their first language, and informed consent may be difficult to obtain compared to Japanese patients. Second, racial and ethnic minorities may be excluded from study populations, as they represent only a small proportion of the patients studied. Although 2.3% of the Japanese population consists of non-Japanese residents,^8^ their enrollment rate in studies may be substantially lower, as medical professionals are likely to face difficulties in obtaining consent or conducting follow-ups due to linguistic and social barriers. In other words, Japanese studies typically include only Japanese patients, and researchers may not be able to identify race and ethnicity in the format designated by the *AMA Manual of Style*.

So, then, should Japanese studies be waived from reporting race or ethnicity? We believe that reporting race and ethnicity from Japan would benefit both the researchers and the study subjects. There are two reasons for this. First, if Japanese studies do not report race or ethnicity, the study may not be eligible to be included or referred to for secondary use. Researchers from other nations may feel uncertain on how to refer to such studies and may question its generalizability towards their clinical or research questions. Such studies might be omitted from secondary studies, such as meta-analysis, which would be detrimental to both the potential citing and cited researchers. Second, excluding non-national patients from studies may not be possible in the future. The rate of foreign nationals living in Japan has been on the increase prior to the COVID-19 pandemic,^8^ and it may become unreasonable to exclude them from studies. The exclusion of such subjects would also be a disadvantage towards the foreign residents living in Japan, as not only their genetic factors but also their unique environmental contexts often linked with race or ethnicity may influence their health status. Therefore, data on foreigners living in Japan would be of necessity in the future, in order to adequately address the health care needs of this population.

Overall, we believe that Japanese studies should also adhere to the *AMA Manual of Style* when referring to the race and ethnicity that is included, even when it only happens to be the Japanese. Such a mindset should benefit both the researchers as well as the subjects and advance the health of populations worldwide. If studies in Japan are to include race and ethnicity in their studies, editors and reviewers of medical and life science journals should consider proposing to authors the reporting of race and ethnicity in accordance with the *AMA Manual of Style*.
